# St. Thomas's Hospital

**Published:** 1916-12-16

**Authors:** 


### NURSES' PAY.

### St. Thomas's Hospital.

The interesting figures given to the Governors of the London Hospital at their last Quarterly Court, and published in The Hospital last week, have naturally led to inquiry elsewhere, and we naturally turned to St. Thomas's Hospital, with its Nightingale Training School, to see what the views of the authorities there might be on what we certainly approve as a move in the right direction.

The figures at the London were £17 for the first year's pay (probationers), £24 for the second year, and £30 for the third year for hospital staff nurses, but £35 for the third year for private nursing staff. The Nightingale authorities have for a long time past recognised three years' training as essential before granting a certificate, and no sister or permanent staff nurse is appointed until certificated, the certificate never being given before the completion of the three years' training, duly tested by periodical examination.

Probationers are of two classes—(i) those who pay for their first year; (ii) those who do not pay and are expected to wait to the end of their fourth year before they are given their certificate.

| No. I. Class. | | No. II. Class. | |
|---|---|---|---|
| 1st year ... ... | — | 1st year ... .. | £10 |
| 2nd year ... ... | £22 | 2nd year ... ... | 20 |
| 3rd year ... ... | 24 | 3rd year ... ... | 22 |
| 4th year, rate of payment to nurses holding a certificate, £35 first six months, £40 second six months. | | 4th year ... ... | 24 |
| | | In their 5th year paid as in No. I. Class as certificated nurses. | |

Ward sisters are paid:—

| | |
|---|---|
| 1st year ... ... ... | £44 per annum. |
| 2nd year ... ... ... | 54 ,, ,, |
| 3rd year ... ... ... | 64 ,, ,, |

There are a number of special sisters' posts which are really well paid, and in these payments we see how fully the Governors acknowledge and recognise the claims of "training" as paramount. Indeed, they make every effort to keep highly skilled teachers available for clinical and theoretical instruction.

Sister in charge of Massage Department and School is paid £150.
,, ,, ,, ,, Nurses' Studies, £100-£150.
,, ,, ,, ,, Preliminary Training School, £80-£90.
,, ,, ,, ,, Mary (Lying-in Ward), £80.
,, ,, ,, ,, Home (Nurses'), £70-£90.
,, ,, ,, ,, Out-patients, £70.
,, ,, ,, ,, *x*-Ray Department, £70-£80.

All these payments are, of course, in addition to full board, lodging, and laundry.

The prestige attaching to a nurse who has been trained at St. Thomas's Hospital is no mean asset for every qualified nurse when she enters the great world to practice her profession. Throughout the hospitals of the country and Empire the reputation of sisters and nurses trained at the Nightingale School is world-wide, and the type is so distinct and so admirable that it is possible for those with a knowledge of the results of training to recognise a graduate of St. Thomas's Hospital when inspecting a hospital where she may occupy a position of responsibility.

The figures we gave a week ago concerning the London Hospital have attracted widespread interest, and we shall be happy to publish particulars of the rates of pay of the nurses in war-time at other important schools, if the authorities will kindly put us in the position to do so by supplying the Editor with the necessary facts.

